# Case report: Long-term management of occlusion after surgical-orthodontic treatment for a patient with drug-induced open bite developed after the onset of schizophrenia

**DOI:** 10.3389/fpsyt.2023.1304215

**Published:** 2023-12-19

**Authors:** Jun-Ichi Takada, Norihisa Higashihori, Chiho Kadota-Watanabe, Tatsuo Kawamoto, Akira Toyofuku, Keiji Moriyama

**Affiliations:** ^1^Department of Maxillofacial Orthognathics, Graduate School of Medical and Dental Sciences, Tokyo Medical and Dental University, Tokyo, Japan; ^2^Division of Orthodontics, Department of Orofacial Sciences, University of California, San Francisco, San Francisco, CA, United States; ^3^Division of Orofacial Functions and Orthodontics, Department of Health Improvement, Kyushu Dental University, Kitakyushu, Japan; ^4^Department of Psychosomatic Dentistry, Graduate School of Medical and Dental Sciences, Tokyo Medical and Dental University, Tokyo, Japan

**Keywords:** surgical-orthodontic treatment, jaw deformity, extra pyramidal symptoms, schizophrenia, drug-induced open bite

## Abstract

**Background:**

Schizophrenia is a major mental disorder, with an estimated incidence of 1%. Since they are sensitive to sensory changes, orthodontic treatment to move teeth should be avoided as aggressively as possible in these patients because of strong concerns about the possibility of causing adverse psychological effects, thus there are few reports on orthodontic treatment for schizophrenia patients. We report a case of severe open bite caused by medication after the onset of schizophrenia, even though the patient’s occlusion had been stable for a long time after surgical orthodontic treatment. Medication control and the use of a minimally invasive orthodontic appliance improved the occlusion without adversely affecting the patient’s mental health.

**Case:**

A 22-year-old woman presented to the clinic with a chief complaint of an anterior open bite. Intraoral findings showed an overbite (vertical overlap of the incisor *teeth*) of −3.0 mm and an overjet (horizontal overlap of the incisor teeth) of −0.5 mm. The preoperative orthodontic treatment included bilateral extraction of the maxillary first premolars. Subsequently, orthognathic surgery was performed to achieve a harmonized skeletal relationship and occlusion. Occlusion was stable for 3 years after surgery. However, 10 years after surgery, the patient returned to the clinic complaining of an anterior open bite (overbite = −4.0 mm). Six years prior to the return, the patient was diagnosed with schizophrenia. We thought that ignoring the patient’s strong desire to treat her open bite might also cause psychological problems; therefore, in addition to medication control, we treated her using a minimally invasive removable orthodontic appliance (retainer with tongue crib). Her anterior open bite improved (overbite, +1.0 mm) to within the normal range.

**Conclusion:**

In this case, medication control was thought to be essential to improve her drug-induced open bite. However, minimally invasive orthodontic treatment, such as the use of a removable appliance, might be helpful in promoting her mental stability as well as for improving occlusion. Careful support is required to obtain information about the patient’s mental state and medications through close cooperation with psychiatrists.

## Introduction

Schizophrenia is a major mental disorder, with an estimated incidence of 1% (1). Recent progress in psychiatric treatments has decreased the number of patients with schizophrenia on admission, and many of them are now receiving psychiatric treatments as outpatients (2). Thus, these outpatients have more opportunities for visiting dental clinics, and some of them could be diagnosed as “jaw deformity” which needs surgical orthodontic treatment. However, patients with schizophrenia are sensitive to sensory changes, and orthodontic treatment to move teeth should be avoided as aggressively as possible because of concerns regarding the possibility of adverse psychological effects (3). However, few reports exist on orthodontic treatment for patients with schizophrenia. Furthermore, some psychiatric medications have a risk of extrapyramidal symptoms (EPS) (4, 5) and may induce an open bite during or after orthodontic treatment, which further complicates the treatment of malocclusion. Because an open bite presents functional problems, such as mastication and pronunciation, leading to a decrease in the patient’s quality of life, there is a high need for its treatment (6–8). Past reports have suggested that open bite can be improved by changing the amount and type of medication taken by patients with schizophrenia, and approaches to improving open bite by controlling medications have been reported (4, 5). The authors also do not recommend invasive orthodontic or prosthetic treatment for patients with drug-induced open bites because, although they may improve temporarily, the condition may recur. However, bluntly refusing a patient’s request to improve his or her open bite can worsen the patient’s mental condition, suggesting that an approach is necessary. This study aimed to report a new treatment approach for patients with schizophrenia who develop a drug-induced open bite.

Here, we report a case of severe open bite, although the patient’s occlusion had been stable for a long time after surgical orthodontic treatment, caused by medication after the onset of schizophrenia, medication control and the use of a minimally invasive orthodontic appliance improved the occlusion without adversely affecting the patient’s mental health.

## Case report

**Figure 1:**
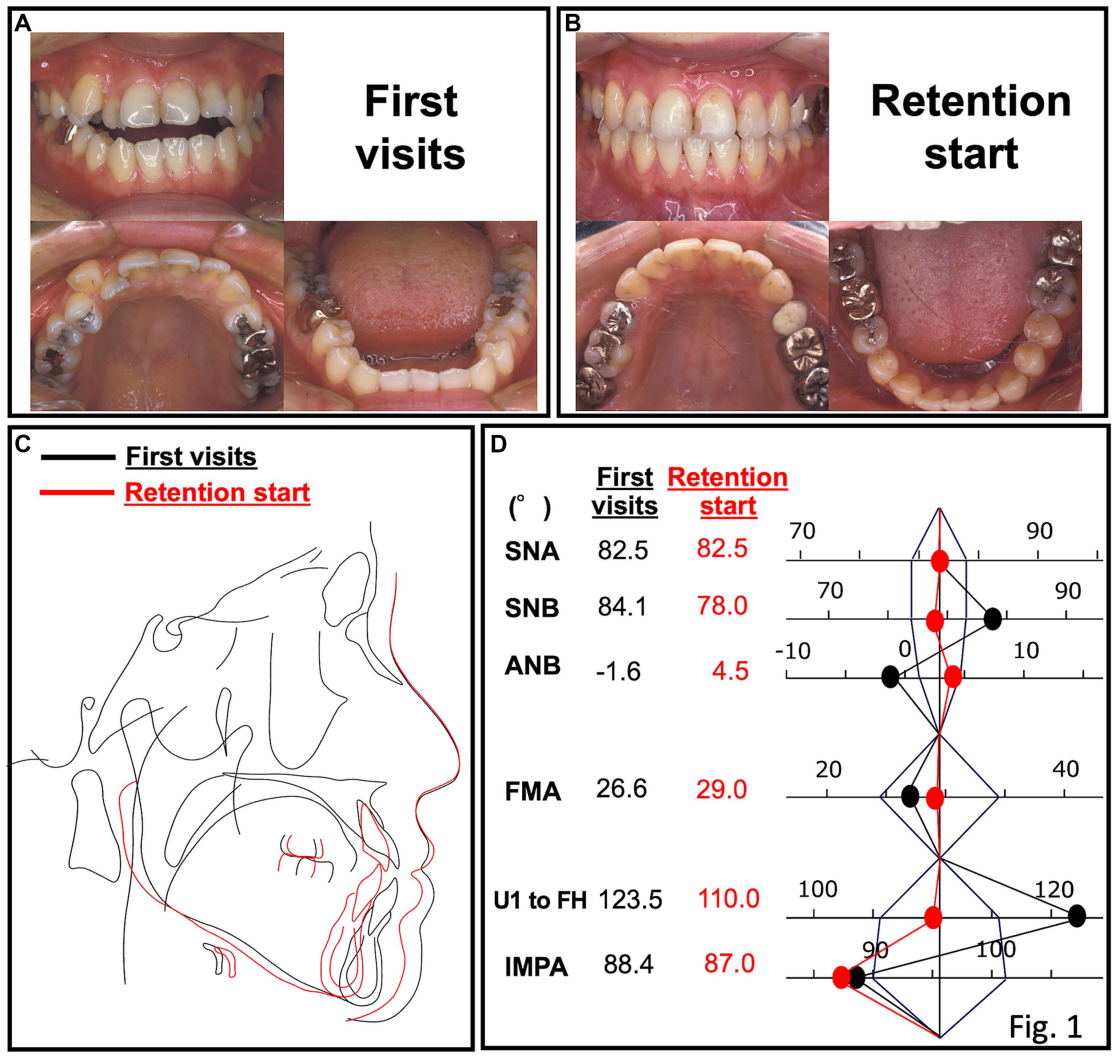
Oral photograph at the first visit **(A)** and the start of the retention period **(B)**. **(C)** Superimposition of cephalograms at the first visit and the start of the retention period. **(D)** Superimposition of polygon analysis at the first visit and the start of the retention period.

**Figure 2:**
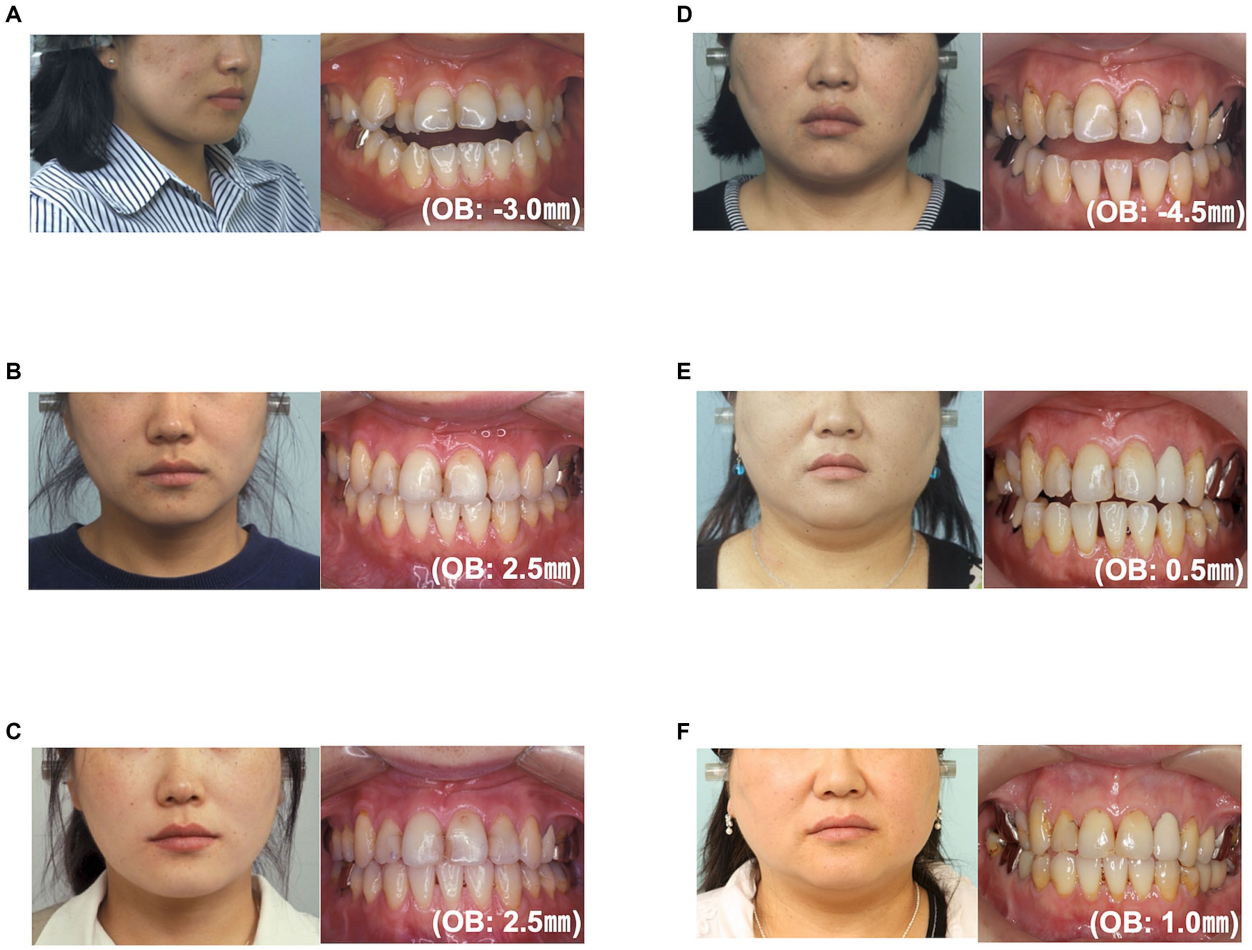
Facial and oral photographs: **(A)** at the first visit; **(B)** at the start of the retention period; **(C)** 1.6 years after retention; **(D)** at the return visit; **(E)** 18.3 years after retention; and **(F)** 20.9 years after retention.

**Figure 3:**
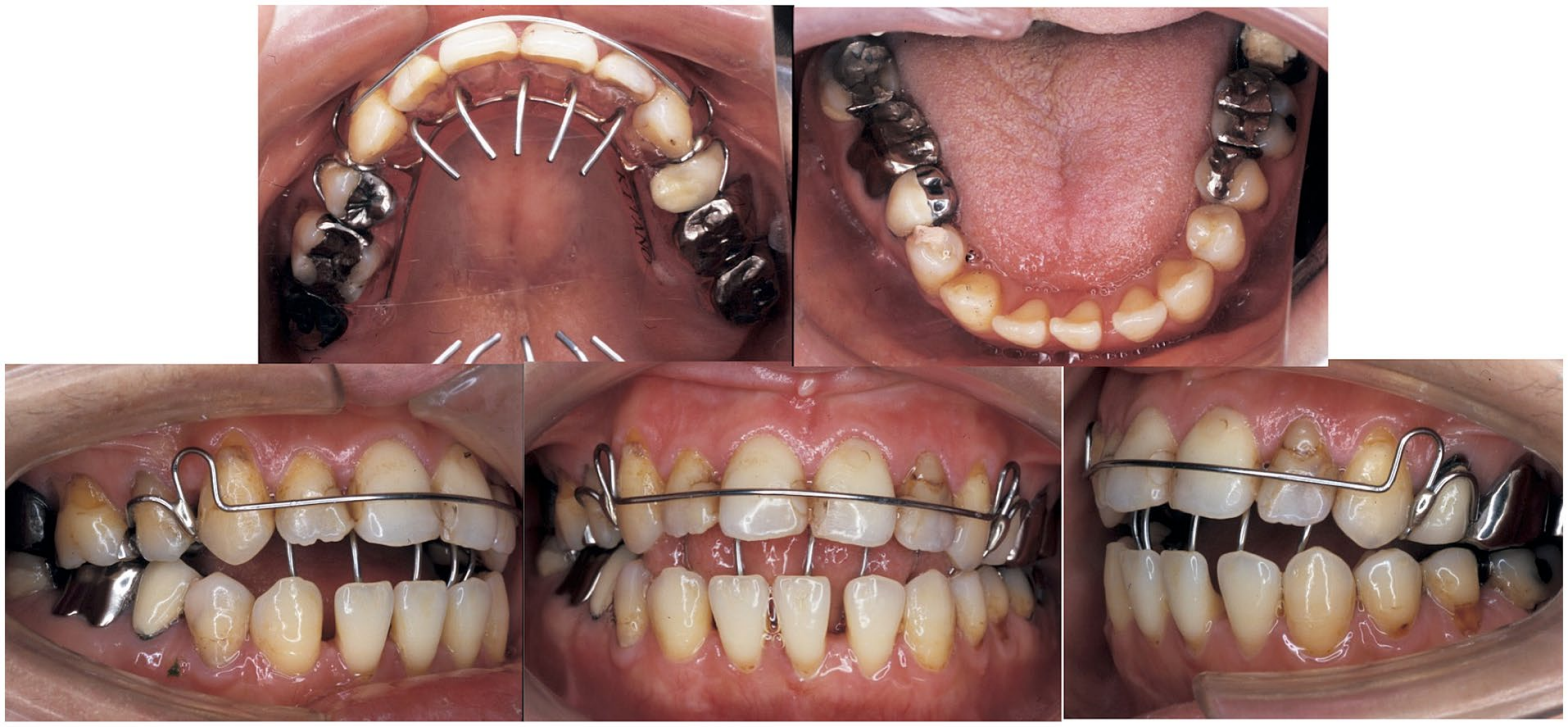
Oral photograph: a retainer with tongue crib was applied.

A 22-year-old woman presented with the chief complaint of an anterior open bite. Intraoral findings revealed a −3.0-mm overbite, −0.5-mm overjet, and a Class III molar relationship. Cephalometric analysis revealed that the mandibular incisor had a protrusion, the maxillary incisor had a buccal inclination, and the mandibular incisor had a lingual inclination (Figure 1). There was no evidence of major medical, family, or psychosocial history. Preoperative orthodontic treatment was performed by extracting the bilateral upper first premolars. Sagittal split ramus osteotomy was subsequently performed at the age of 26 years to achieve a skeletal relationship and occlusion. Surgical orthodontic treatment was completed at the age of 29 years and transitioned to a retention period. The occlusion was stable for 3 years after surgery. However, she was diagnosed with schizophrenia at 34 years of age. The patient was administered haloperidol (5 mg/day), olanzapine (5 mg/day), or fluvoxamine (100 mg/day). Ten years after the start of the retention period, the patient returned to our department complaining of an anterior open bite (overbite: −4 mm) at 40 years of age. After consultation with her psychiatrist, her prescription was changed to aripiprazole (12 mg/day), Olanzapine (2.5 mg/day), and fluvoxamine (50 mg/day). We also informed her to use a retainer with a tongue crib to reduce the effect of tongue thrust. Her medication was decreased to aripiprazole (9 mg/day) and fluvoxamine (50 mg/day) at 41 years of age, and was again reduced to aripiprazole (3 mg/day) and fluvoxamine (50 mg/day) at 43 years of age. At 44 years of age, the dosage was decreased to aripiprazole (3 mg/day) and fluvoxamine (50 mg/day). At 47 years of age, there was a decrease in the open bite, and at 50 years of age, there was a further decrease and improvement in the open bite (Table 1). The patient’s open-bite gradually improved (Figures 2, 3). The patient reported that the use of the device made her feel mentally reassured. Currently, there have been no cases of dental relapse in this patient, and no adverse psychological effects have been observed during treatment.

**Table 1:** Relationship between drug dosage and overbite.

	First visit	Start of the retention period	1.6 years after retention	Diagnosis of schizophrenia	Return visit	Tongue crib start	18.3 years after retention	20.9 years after retention
Age (years)	22	29	30	34	40	41	47	50
Overbite (mm)	−3.0	2.5	2.5	–	−4.5	−4.5	0.5	1.0
Haloperidol (mg/day)	–	–	–	5	–	–	–	–
Aripiprazole (mg/day)	–	–	–	–	12	9	3	3
Olanzapine (mg/day)	–	–	–	5	2.5	–	–	–
Fluvoxamine (mg/day)	–	–	–	100	100	50	30	25

For ethical considerations, we have written the report in such a way that the patient cannot be identified, and written consent was obtained from the patient for the publication of this report. This case report is based on the information obtained from the patient herself, as detailed information could not be obtained from her psychiatrist because of privacy concerns.

## Discussion

Orthodontic treatment usually begins in childhood and adolescence. Predicting the onset of schizophrenia in such patients before initiating orthodontic treatment is extremely difficult. At 22 years of age, our patient was diagnosed with skeletal mandibular prognathism and underwent orthodontic treatment. The surgical orthodontic treatment was completed at 29 years of age. However, at 5 years post-retention, she was diagnosed with schizophrenia and started on D2 blockers; at 11 years post-retention, she developed a severe open bite. Because the patient had not visited our clinic for 6 years since her diagnosis of schizophrenia, the details of her oral condition were unclear. However, the patient returned to our clinic complaining that she had gradually lost her ability to bite and strongly wanted orthodontic treatment again. During follow-up, the patient’s overbite was measured at each visit, and occlusal contact was checked using an articulating paper. In addition, if necessary, a lateral cephalogram was obtained and compared with previous cephalograms to confirm the results.

Previous studies have reported that it is extremely difficult to manage fixed appliance orthodontic treatment in patients with schizophrenia, especially those receiving antipsychotic medications, because such patients tend to have dry mouth and a higher prevalence of caries, gingivitis, and periodontal disease (9). Previous reports have shown that psychological stress can decrease tooth movement during orthodontic treatment (10). Changes in the occlusion of patients with schizophrenia increase the risk of worsening psychiatric problems (11). Thus, considering the possibility that a fixed-type appliance (i.e., multi-bracket appliance) would put too much psychological stress to this patient, a removable appliance (i.e., retainer with tongue crib) was selected.

In general, skeletal and dental relapses occur within 1 year after orthognathic surgery or retention, respectively (12). Interestingly, the patient had a stable occlusion until 3 years after retention but developed a severe open bite without skeletal relapse. One of the causes of relapse (open bite) is oral habits (e.g., finger sucking, low tongue, tongue thrusting) (13); however, in these cases, relapse is seen gradually after surgery or after retention begins. Thus, it is difficult to imagine a sudden onset of relapse a few years later, as in the present case. Additionally, the results of the lateral cephalometric diagnostic test in this case showed a dental, but not a skeletal, relapse after the start of retention and at the development of an open bite (data not shown). From this perspective, the relapse observed in this case may have been caused by a combination of other factors.

Nakamura (4) reported that an extrapyramidal blockade with haloperidol, an antipsychotic, can produce dystonia, dyskinesia, and other involuntary movements that trigger open-bite symptoms. They reported this as a drug-induced open bite. First-generation antipsychotics, including the centrally acting dopamine receptor blockers, haloperidol and phenothiazine neuroleptics, are known to be antipsychotics associated with EPS. After switching from haloperidol (5 mg/day) to the dopamine partial agonist aripiprazole (12 mg/day), her open bite gradually improved. Furthermore, the use of a removable appliance (retainer with tongue crib) was thought to contribute for improving the open bite in combination with medication and dosage control.

In addition, a previous report showed that dystonia of the masticatory muscles can be one of the causes of an open bite, which was also considered one of the causes in this case (4). Other possible causes include tongue dystonia and tongue protrusions caused by dyskinesia. However, previous studies have not elucidated the detailed mechanisms underlying drug-induced open bites (14). Thus, at the time of medication change, the patient was instructed to use an orthodontic removable retention appliance with tongue cribs to prevent tongue protrusion. The patient reported that using this device made her feel mentally reassured, suggesting that it promoted mental stability. The patient seemed to have difficulty in waiting for the reduction of medication for a long time. These findings suggest that not only medication control but also the use of a removable retentive appliance to prevent tongue protrusion may be helpful in these cases.

Our case suggests that careful support is needed to obtain information about a patient’s mental state and medications through close cooperation with psychiatrists. Moreover, detecting drug-induced open bite from the patient’s systemic and intraoral findings and eliminating its cause are considered important not only for orthodontic treatment, but also before any dental treatments. It was suggested that not only patients with jaw deformities, such as this patient, but also patients with orthodontic treatment should be presented with an “informed consent” with regard to psychiatric medication, and they must be informed that psychiatric medications may affect occlusal conditions, especially in cases of surgical-orthodontic treatments.

In general, patients with schizophrenia are highly sensitive to sensory changes; thus, orthodontic treatment that changes the position of the teeth may cause mental ataxia and should be avoided. Furthermore, irreversible dental treatment (e.g., prosthetic or orthodontic) for an open bite caused by drugs for schizophrenia is contraindicated because the bite improves temporarily but is affected again by the drugs (14). This is a new approach for the treatment of drug-induced open bites that is meaningful for both psychiatrists and orthodontists because it is based on the patient’s desire to cure his or her occlusion through collaborative treatment between psychiatrists and orthodontists. The patient herself was willing to use the appliance and commented that it helped stabilize her psychologically, which may have led to a reduction in medication dosage. Thus, the strength of this study lies in its case report on the synergistic effect of the use of a removable orthodontic appliance and medication management to achieve psychological and occlusal stability.

Nevertheless, this study has some limitations. First, the patient did not wish to communicate with a psychiatrist; therefore, the details before and after the disease onset are not clear. Secondly, because this was a single case, it is unclear whether the same improvement can be achieved in other similar cases. Therefore, a close collaboration with psychiatrists is necessary to explore other similar cases.

## Conclusion

In this case, medication control was thought to be essential to improve the patient’s drug-induced open bite; however, minimally invasive orthodontic treatment, such as the use of a removable appliance, might be helpful in promoting her mental stability as well as for improving occlusion. Careful support is required to obtain information about the patient’s mental state and medications through close cooperation with psychiatrists.

## Data availability statement

The original contributions presented in the study are included in the article/supplementary material, further inquiries can be directed to the corresponding author.

## Ethics statement

Written informed consent was obtained from the individual(s) for the publication of any potentially identifiable images or data included in this article.

## Author contributions

J-IT: Writing – original draft, Writing – review & editing. NH: Writing – original draft, Writing – review & editing. CK-W: Writing – review & editing. TK: Writing – review & editing. AT: Writing – review & editing. KM: Writing – review & editing.
