# Supplementary material for: Case report: Long-term management of occlusion after surgical-orthodontic treatment for a patient with drug-induced open bite developed after the onset of schizophrenia
Source: Front Psychiatry. 2023 Dec 19;14:1304215. doi: 10.3389/fpsyt.2023.1304215 (PMC10763243; doi:10.3389/fpsyt.2023.1304215)
Supplement: Supplementary file 1 [file Data_Sheet_1.pdf]

CARE
case report guidelines

CARE Checklist of information to include when writing a case report

(cc) BY-NC-ND

✓

Topic	Item	Checklist item description	Reported on Line
**Title**	1	The diagnosis or intervention of primary focus followed by the words "case report"	55
**Key Words**	2	2 to 5 key words that identify diagnoses or interventions in this case report, including "case report"	171-172
**Abstract (no references)**	3a	Introduction: What is unique about this case and what does it add to the scientific literature?	80-84
	3b	Main symptoms and/or important clinical findings	84-88
	3c	The main diagnoses, therapeutic interventions, and outcomes	96-102
	3d	Conclusion—What is the main "take-away" lesson(s) from this case?	104-106
**Introduction**	4	One or two paragraphs summarizing why this case is unique (**may include references**)	131-149
**Patient Information**	5a	De-identified patient specific information.	160-200
	5b	Primary concerns and symptoms of the patient.	180-186
	5c	Medical, family, and psycho-social history including relevant genetic information	165-166
	5d	Relevant past interventions with outcomes	186-200
**Clinical Findings**	6	Describe significant physical examination (PE) and important clinical findings.	224-276
**Timeline**	7	Historical and current information from this episode of care organized as a timeline	180-196
**Diagnostic Assessment**	8a	Diagnostic testing (such as PE, laboratory testing, imaging, surveys).	460-465
	8b	Diagnostic challenges (such as access to testing, financial, or cultural)	201-207
	8c	Diagnosis (including other diagnoses considered)	336-465
	8d	Prognosis (such as staging in oncology) where applicable	N/A
**Therapeutic Intervention**	9a	Types of therapeutic intervention (such as pharmacologic, surgical, preventive, self-care)	474-476
	9b	Administration of therapeutic intervention (such as dosage, strength, duration)	186-197
	9c	Changes in therapeutic intervention (with rationale)	466-485
**Follow-up and Outcomes**	10a	Clinician and patient-assessed outcomes (if available)	512-514
	10b	Important follow-up diagnostic and other test results	224-276
	10c	Intervention adherence and tolerability (How was this assessed?)	198-201
	10d	Adverse and unanticipated events	198-201
**Discussion**	11a	A scientific discussion of the strengths AND limitations associated with this case report	514-523
	11b	Discussion of the relevant medical literature **with references**.	211-512
	11c	The scientific rationale for any conclusions (including assessment of possible causes)	527-534
	11d	The primary "take-away" lessons of this case report (without references) in a one paragraph conclusion	527-534
**Patient Perspective**	12	The patient should share their perspective in one to two paragraphs on the treatment(s) they received	512-514
**Informed Consent**	13	Did the patient give informed consent? Please provide if requested	Yes ☑ No ☐
